# Application of Andersen–Newman model to assess cataract surgery uptake among older Australian women: findings from the Australian Longitudinal Study on Women’s Health (ALSWH)

**DOI:** 10.1007/s40520-022-02091-2

**Published:** 2022-02-19

**Authors:** Mitiku Teshome Hambisa, Xenia Dolja-Gore, Julie Byles

**Affiliations:** 1 Centre for Women’s Health Research, University of Newcastle, Callaghan, NSW 2308 Australia; 2 Centre for Clinical Epidemiology and Biostatistics, University of Newcastle, Callaghan, NSW 2308 Australia; 3 School of Public Health, Haramaya University College of Health and Medical Sciences, P. O. Box 235, Harar, Ethiopia

**Keywords:** Cataract surgery, Older women, Health service utilization, Increasing age

## Abstract

**Introduction:**

Although Cataract Surgery Rate is increasing, the availability of surgery is outstripped by the increasing number of cataract cases as populations age.

**Aim:**

The study aimed to identify factors associated with cataract surgery uptake in terms of predisposing, enabling, and need factors in very old Australian women.

**Method:**

This study used ALSWH data included 6229 women aged 79–84 to 85–90 years. Women were asked whether they had undergone eye surgery (including cataracts) three years prior to each survey. Generalised estimating equation modelling was used to determine factors associated with these surgeries.

**Result:**

At baseline (2005), more than half of the participants either had undergone surgery (43.5%) or had unoperated cataracts (7.6%). Increasing age (AOR = 1.11, 95% CI = 1.07, 1.15) and being current or ex-smokers (AOR = 1.15, 95% CI = 1.03, 1.29) were associated with higher odds of cataract surgery (predisposing factors). Women who had private health insurance had 27% higher odds of having surgery (AOR = 1.27, 95% CI = 1.16, 1.39) (enabling factor). Need factors of more General Practitioner visits (AOR = 1.16, 95% CI = 1.09, 1.25) and skin cancer (AOR = 1.09, 95% CI = 1.01, 1.17) also increased the odds of cataract surgery. Women who had no difficulty seeing newspaper print were more likely to have had cataract surgery (AOR = 1.35, 95% CI = 1.23, 1.48).

**Conclusion:**

Need factors are the major drivers of cataract surgery; however, predisposing and enabling factors also play a role, including access to private health insurance. This finding indicates some inequity regarding access to cataract surgery in the Australian setting.

## Introduction

Age-related cataract is the principal cause of visual impairment and blindness among older people in the world [1–3], with cataract surgery being one of the most performed surgical procedures, safely, and the only effective intervention to treat cataracts [4–6]. For economically developed countries, the Cataract Surgery Rates are estimated to range from 4,000 to 10,000 surgeries per million people per year. However, Cataract Surgery Rates vary greatly from country to country and for different subpopulations within countries [7].

In Australia, one in three people older than 65 years [8] and over 70% of people aged 80 years and above have clinically significant cataract [8]. Furthermore, Australia’s number of older people with cataracts is estimated to increase from 1.7 million in 2001 to 2.7 million people in 2021, primarily due to the impact of population ageing [9].

Cataract surgery remains the most performed surgical procedure and the second most common elective surgery in Australia [10, 11], with more than 250,000 people undergoing cataract surgery each year [12]. Early in the course of the disease, prescription eyeglasses and brighter reading lights may help for a while [13, 14], but as people age, cataract is likely to advance and eventually, cataract surgery is the only proven effective treatment [5, 13, 14]. However, many people in Australia with age-related cataracts are waiting for surgery, with surgery rates not keeping pace with the rising prevalence of cataracts in our ageing population [15–18].

The waiting time for cataract surgery in Australia can take up to 3 years from referral to treatment especially for public-funded Medicare beneficiaries, where the last 12 months spent on the surgery waiting list for pre-treatment ophthalmologic evaluation [11, 19]. The waiting lists are longer in more socio-economic disadvantaged areas [20]. Moreover, in a recent survey of ophthalmologists, waiting times were less for patients accessing the private health system than for those waiting to be treated in the public sector [21]. While waiting, these people are at risk of injury, fall, fracture, car crash [16, 22, 23] and other adverse outcomes [24]. For instance, a study in the United Kingdom showed that reducing the waiting time for surgery from 12 months to 1 month reduced the risk of fall by 34% [25].

Cataract surgery rates are likely to be affected by both the prevalence of cataract, and the factors that predispose to this condition as well as by the factors affecting access to surgery [7, 26, 27]. Both factors are strongly affected by socio-economic determinants of health [7, 28]. Cataract is associated with multiple factors that accumulate across the life course including exposure to ultraviolet light, smoking, poor nutrition, diabetes, and severe dehydration [29, 30].

Earlier studies have identified factors associated with cataract surgery including increasing age [27, 31], vision problem [27], diabetes [27, 32], private health insurance [31], estrogen replacement therapy in women participants [27, 33], self-rated health [34, 35], private employer-based health insurance (lower rate surgery) [31], smoking [27, 36], and educational level [37].

In a recent review [38], that assessed factors associated with cataract surgery uptake, it was concluded that demographic and socio-economic characteristics, such as age [38], gender, wealth [38, 39] (particularly the capacity to pay) [26], and need factors such as health status, poor vision [39, 40], and knowledge of surgery [39] were found to be associated with cataract surgery utilization [38]. Regarding gender, sex was examined as an influencing factor in many studies, where significant differences were found in cataract surgery uptake between female and men in which the utilization was found to be higher among men [38, 40, 41].

While several cross-sectional studies have assessed factors associated with and barriers for cataract surgery uptake [42–45], only few studies [31] longitudinally examined factors associated with cataract surgery over time, particularly in very advanced aged population in terms of predisposing, enabling, and need factors.

Also, previous studies of cataract surgery put great emphasis on the identification of risk factors for surgery; but what is less known is what factors affect cataract surgery services utilization in terms of predisposing, enabling, and need factors. These factors were identified in the Andersen–Newman behavioural model of health service utilization. The Andersen–Newman’s model assumes to achieve equitable health services distribution while acknowledging societal changes over time and addressing the variability of health service use with time when services were not similar across vulnerable populations [46, 47]. The model suggests that there is a series of conditions that lead to health service utilization that includes need level (mostly illness or disease-related factors, such as cataract, poorer vision in case of cataract surgery) which could be considered the primary reason for a person to seek healthcare, predisposing characteristics (mainly demographic factors, such as genetic, age, and gender) that could have medium importance, and the last is enabling factors which are related to resources with regard to equitable healthcare use [46, 47]. Lin, Ma et al. [26] applied this model to identify determinants of cataract surgery uptake in China and 19 sub-Saharan African countries and found that the enabling factor of capacity to pay in China and the availability of cataract service provision in sub-Saharan African countries were associated with cataract surgery uptake [26].

This study applies the Andersen–Newman behavioural model of health service utilization to identify factors associated with cataract surgery over 6-year of follow-up in terms of predisposing, enabling, and need factors among a large cohort of women in their 80 s. Cataract is more common in older women [8] and places their daily activities and ability to live independently at risk.

## Methods

### Sample

Data for this study were drawn from the 1921–26 cohort of the ALSWH, a nationally representative prospective longitudinal study of Australian women (http://www.alswh.org.au) [48, 49]. The women were sampled from the Australian Medicare database and were first surveyed in 1996 when the women were 70–75 years. Since that time, participating women have been surveyed every 3 years from 1996 to 2011 (surveys 1–6), and 6 monthly after that [48, 50]. This study uses survey data from Survey 4 (ages 79–84) to Survey 6 (85–90).

### Outcome variable

#### Cataract surgery (self-reported)

For each of surveys 2, 3, 4, 5 and 6, the questionnaires asked if the women had eye surgery (including cataract) 3 years prior to that survey, and for surveys 4, 5 and 6 if they had been diagnosed with or treated for cataract in the past 3 years of the survey. For each survey, the response to each question was categorised as “yes”, “no”, or “missing”. For this analysis, once a woman had answered “yes” to cataract surgery in one survey, she was considered to have had at least one cataract operation for all future surveys (enduring). Cataract was not considered as an enduring condition as it can be corrected by surgery and can reappear in the contralateral eye. We excluded participants who had eye surgery prior to survey 2. We also used survey 4 as a baseline to include cataracts in the analysis.

### Explanatory variables

Each survey included measures of, predisposing factors of age, education, smoking status, country of birth, alcohol drinking status; enabling factors of area of residence (classified using ARIA + [51, 52]), having private health insurance; and primary need factors including cataract, difficulty seeing newspaper print, even with glasses. Andersen model also suggests three components that influence health service utilization one of which is the need level which is mostly illness or disease-related factors such as cataract and poorer vision in case of cataract surgery which is considered the primary reason for a person to seek health service use recognizing variability of health service utilization with societal changes and time [46, 47]. Additional need factors included general health, physical function score, social function score, general practitioner (GP) visit, driving, and chronic medical conditions, such as skin cancer, diabetes, hypertension, fall, hormone replacement therapy. These factors were based on the Andersen–Newman behavioural model of health service use [46, 47], and on the basis of the previous literature with regard to factors associated with cataract surgery [27, 31, 49, 53]. All the independent variables were taken from ALSWH surveys 4 to 6 apart from hormone replacement therapy (measured at surveys 1& 3), alcohol use status measured at surveys 3 and 6), smoking status (measured at survey 2), and polypharmacy which was measured at survey 3.

### Statistical analysis

After merging data for surveys 4, 5, and 6, we examined response patterns for cataract surgery across survey waves. Based on baseline data, explanatory variables were then screened for correlation with each other using Spearman’s statistical test, and bivariate associations between explanatory variables and cataract surgery were investigated using chi-square test (categorical) and *t* test (continuous). All variables with *p* < 0.25 were considered for multivariable analysis. Generalised estimating equation (GEE) modelling was conducted to account for the repeated measures of cataract surgery over three timepoints (6 years of follow-up) based on the Liang and Zeger approach [54].

The following explanatory variables were correlated with other variables (correlation coefficient > 0.3 considered) and excluded from the analysis: physical function (PF) score was correlated with general health, driving, fall, hypertension, and GP visit; social function (SF) score correlated with driving, GP visit and self -rated health; education correlated with private insurance. The variables of marital status, area of residence, country of birth, education, Body Mass Index (BMI), and stroke were all non-significant on bivariate screening and omitted from the multivariable modelling process.

GEE models were constructed applying binary distribution of the outcome variable, with a logit link function and autoregressive correlation structure as cataract surgery was measured over three-timepoints with equal 3-yearly time intervals. Initially, separate sets of models were constructed for the predisposing, enabling and need factors based on the Andersen-Newman behavioural model for health care utilization [46]. Sets of significant variables for each of these factors were then entered into nested GEE models as follows:

*Model 1*: considered predisposing factors: age, smoking status, alcohol consumption.

*Model 2*: included significant factors from model 1, plus enabling factors.

*Model 3***:** included significant factors from model 2, plus need factors.

*Model 4***:** Final parsimonious nested model.

Time was included in all four models in accordance with the data structure. The results were expressed as odds ratios (OR) with 95% confidence intervals.

### Ethical approval

The ALSWH has continuous ethical approval from the Human Research Ethics Committees (HREC) of the University of Newcastle (reference H-076-0795) and the University of Queensland (reference 2004000224).

## Results

Of the 12,432 women who started the study in 1996, 2289 women had died prior to survey 4, and 525 had withdrawn from the study, stating they were “too frail”. Of the remainder, 7158 (74%) women completed survey 4. However, 121 of these women had missing data for cataract surgery at survey 4, and another 245 were missing information for cataract surgery for all surveys 4–6. A further 493 had prior eye surgery (including cataract) more than 3 years before the commencement of the ALSWH (1996), as reported on Survey 2. After excluding these women, a total of 6299 women remained in the baseline for subsequent longitudinal analysis. Of these women, 4711 remained in the study at survey 5 (1036 did not respond; 552 deceased), and 3241 remained in the study at survey 6 (1634 did not respond; 1424 deceased). The main cause for attrition from survey one was due to death [55].

**Fig. 1:**
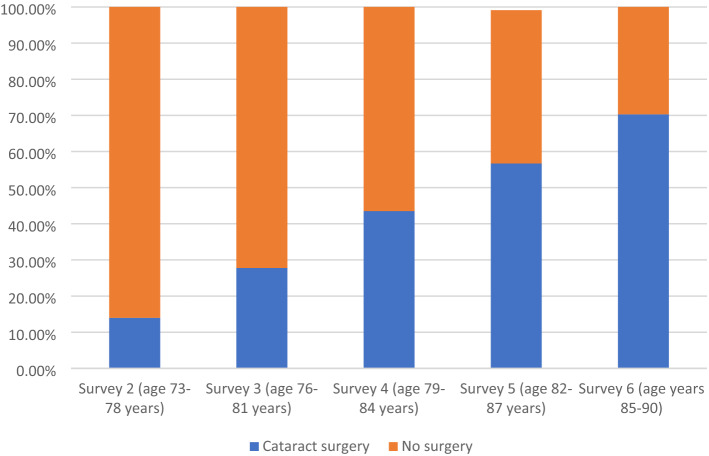
shows the proportion of women reporting cataract surgery at each survey from survey 2 onwards (age 73–90 years)

Looking at the proportion of women reporting cataract surgery at each survey for survivors from survey 2 onwards (either reporting on that survey or on an earlier survey), the cataract surgery rate increased with age, being 14.0% at survey 2 (age 73–78 years) and 70.3% at survey 6 when they were aged 85–90 years (Fig. 1).

Table 1 shows the numbers of women who had cataract surgery by survey 4 (either reporting on that survey or an earlier survey) and the socio-demographic characteristics, predisposing, enabling, and need factors for cataract surgery uptake of the ALSWH participants at baseline. By survey 4, 44% of the women had undergone cataract surgery. Another 7.62% had reported cataract on or prior to survey 4, but not reported cataract surgery. In total, over 51% of the participants either had undergone surgery or had unoperated cataract by Survey 4 (Table 1).

**Table 1:** Baseline characteristics for women who had one or more cataract surgeries by survey 4 (age 79–84 years)

Baseline explanatory variables according to Andersen–Newman behavioural model	Total *N* (%)	Participants according to whether they had cataract surgery or not by Survey 4 (*N* = 6299)		*P* value
		Yes 2741 (43.51%)	No 3558 (56.49%)	
Predisposing Factors				
Age in years	(Mean, SD)	80.87 (1.43)		0.0002
Marital status				0.294
Partnered	2314 (37.0)	953 (30.5%)	1361 (43.4%)	
Non-partnered	3946 (63.0)	2173 (69.5%)	1773 (56.6%)	
Educational Qualification				0.3636
No formal qualifications	1774 (29.47)	751 (28.8)	1023 (30.0)	
School Certificate	2402 (39.90)	1072 (41.1)	1330 (39.0)	
High School Certificate	800 (13.29)	334 (12.8)	466 (13.7)	
Trade/college or University	1044 (17.34)	454 (17.4)	590 (17.3)	
Smoking status				0.0298
Never-smoker	3803 (66.47)	1631 (64.9)	2172 (67.7)	
Ex-smoker/current smoker	1918 (33.53)	881 (35.1)	1037 (32.3)	
Alcohol consumption				0.2746
Drinker	2330 (39.08)	1046 (40.1)	1284 (38.3)	
Non-drinker	3632 (60.92)	1564 (59.9)	2068 (61.7)	
Country of birth				0.8816
Australian born	4747 (79.71)	2072 (79.9)	2675 (79.6)	
Other English-Speaking Country	754 (12.66)	322 (12.4)	432 (12.8)	
Europe/Asia/Other	454 (7.62)	199 (7.7)	255 (7.6)	
Enabling Factors				
Accessibility/Remoteness Index for Australia (ARIA +)				0.6505
Major cities	2817 (44.73)	1232 (45.0)	1585 (44.6)	
Inner regional	2330 (37.00)	998 (36.4)	1332 (37.4)	
Outer regional/Remote/very remote	1151 (18.27)	511 (18.6)	640 (18.0)	
State				0.0616
New South Wales (NSW)	2163 (34.34)	983 (35.9)	1180 (33.2)	
Victoria (VIC)	1568 (24.89)	646 (23.5)	922 (25.9)	
Queensland (QLD)	1154 (18.32)	507 (18.5)	647 (18.2)	
South Australia (SA)	620 (9.84)	249 (9.1)	371 (10.4)	
Western Australia (WA)	495 (7.86)	218 (8.0)	277 (7.8)	
TAS/ACT/NT	299 (4.75)	138 (5.0)	161 (4.5)	
Private health insurance				0.0086
Yes	1654 (26.54)	797 (29.3)	857 (24.4)	
No	4579 (73.46)	1923 (70.7)	2656 (75.6)	
Primary Need factors				
Cataract				< 0.0001
Yes	2018 (32.30)	1542 (56.5)	476 (13.5)	
No	4230 (67.70)	1189 (43.5)	3041 (86.5)	
Difficulty seeing newspaper print, even with glasses				0.0972
Yes	1389 (22.36)	576 (21.3)	813 (23.1)	
No	4823 (77.64)	2123 (78.7)	2700 (76.9)	
Other Need factors				
Physical function score	(Mean, SD)	53.04 (28.2)		< 0.0001
Social function score	(Mean, SD)	75.96 (27.12)		< 0.0001
General health				< 0.0001
Good	4293 (68.48)	1772 (64.9)	2521 (72.3)	
Poor	1976 (31.52)	960 (35.1)	1016 (28.7)	
Diabetes				0.0206
Yes	722 (11.56)	345 (12.6)	377 (10.7)	
No	5526 (88.44)	2386 (87.4)	3140 (89.3)	
Fall				0.0727
Yes	1440 (23.60)	654 (10.72)	786 (12.88)	
No	4661 (76.40)	1990 (32.62)	2671 (43.78)	
Hypertension				0.0170
Yes	3529 (56.23)	1584 (57.9)	1945 (54.9)	
No	2747 (43.77)	1150 (42.1)	1597 (45.1)	
Skin cancer				0.0723
Yes	1559 (24.95)	712 (26.1)	847 (24.1)	
No	4689 (75.05)	2019 (73.9)	2670 (75.9)	
Hormone replacement therapy				0.0021
Yes	5165 (89.55)	300 (11.9)	303 (9.3)	
No	603 (10.45)	2227 (88.1)	2938 (90.7)	
GP visit				< 0.0001
4 or less (low)	2322 (37.53)	872 (14.09)	1450 (23.44)	
5 or more (high)	3865 (62.47)	1820 (29.42)	2045 (33.05)	
Driving				0.7339
Yes	2932 (49.21)	1260 (49.0)	1672 (49.4)	
No	3026 (50.79)	1314 (51.0)	1712 (50.6)	

Missing value ranges from 0.01 to 8%. and ‘*n*’ sizes may vary due to missing data

The mean age (± standard deviation) was 80.87 (± 1.43) years at baseline. Most (63.0%) were non-partnered, 39.9% had school certificate level of education, 13.3% had high school certificate, and 17.3% had trade/college or University degree, 33.5% were ex-smoker/current smokers, and 44.7% live in metropolitan, 37.0% live in inner regional, 18.3% live in remote, very remote, and outer regional Australia.

In terms of need factors, 476 (7.62%) had reported unoperated cataract, 22.4% had difficulty seeing newspaper print even with glasses, more than 68% had excellent, very good or good general health, 26.5% had private health insurance, around 90% had used hormone replacement therapy, 25.0% had skin cancer, and 62.8% had high GP visit in the past 12 months of the survey.

In multivariable nested models (Table 2, model 4), increasing age was associated with higher odds of cataract surgery (AOR = 1.11, 95% CI = 1.07, 1.15), and the odds of cataract surgery increased with time (ageing). Women who were current or ex-smokers had higher odds of cataract surgery (AOR = 1.15, 95% CI = 1. 03, 1.29) compared with never smokers (predisposing factors). From the enabling factors, women who had private health insurance had 27% higher odds of having cataract surgery (AOR = 1. 27, 95% CI = 1.16, 1.39) compared to those who didn’t have private health insurance.

**Table 2:** Factors associated with cataract surgery among older Australian women (age 79–84 to 85–90 years) according to predisposing, enabling and need factors (Anderson behavioural Model), 2021

Explanatory variables based on Anderson–Newman healthcare utilization behavioural model	Model 1 (predisposing)	Model 2 (predisposing + enabling)	Model 3 (predisposing + enabling + need)	Model 4 (Final nested model)
	Adjusted OR (95% Confidence Intervals)			
Intercept	0.04 (0.001, 0.007)	0.001 (0.01, 0.04)	0.001 (0.002, 0.003)	0.001 (0.001, 0.007)
Time Survey 4 (2005, age 79–84 years) (ref)	1.0	1.0	1.0	1.0
Survey 5 (2008, age 82–87 years)	1.86 (1.78, 1.94)	1.86 (1.78, 1.95)	1.83 (1.74, 1.93)	1.82 (1.73, 1.91)
Survey 6 (2011, age 85–90 years)	3.35 (3.13, 3.59)	3.76 (3.15, 3.61)	3.40 (3.14, 1.67)	3.39 (3.14, 3.66)
Predisposing Factors				
Age in years**	1.09 (1.06, 1.14)	1.10 (0.99, 1.06)	1.11 (1.07, 1.15)	1.11 (1.07, 1.15)
Smoking status**				
Never-smoker	1.0	1.0	1.0	1.0
Ex-smoker/current smoker**	1.15 (1.03, 1.27)	1.15 (1.04, 1.28)	1.14 (1.02, 1.28)	1.15 (1.03, 1.29)
Enabling factors				
Private health insurance**				
No		1.0	1.0	1.0
Yes		1.24 (1.14, 1.35)	1.27(1.16, 1.39)	1.27 (1.16, 1.39)
Primary Need factor				
Difficulty seeing newspaper print, even with glasses**				
Yes			1.0	1.0
No			1.35 (1.22, 1.48)	1.35 (1.23, 1.48)
Other Need factors				
General health**				
Good			1.0	1.0
Poor			1.22 (1.13, 1.32)	1.21 (1.12, 1.30)
Diabetes				
No			1.0	
Yes			1.05 (0.92, 1.19)	
Skin cancer**				
No			1.0	1.0
Yes			1.08 (1.01, 1.16)	1.09 (1.01, 1.17)
Hormone replacement therapy**				
No			1.0	1.0
Yes			0.82 (0.69, 0.98)	0.82 (0.69, 0.98)
Fall				
No			1.0	
Yes			1.06 (0.99, 1.13)	
Hypertension				
No			1.0	
Yes			0.96 (0.89, 1.03)	
Driving				
No			1.0	1.0
Yes			0.93 (0.85, 1.01)	0.92 (0.84, 1.01)
GP visit**				
Low			1.0	1.0
High			1.18 (1.09, 1.26)	1.16 (1.09, 1.25)

**Significant at p < 0.05

Pertaining to primary need factors, women with cataract (*P* = 0.0001) at baseline and women who had no difficulty seeing newspaper print were more likely to have had cataract surgery (AOR = 1. 35, 95% CI = 1.23, 1.48) compared to those who had difficulty seeing newspaper print.

Five or more GP visits in the past 12 months of the survey were associated with higher odds of cataract surgery (AOR = 1. 16, 95% CI = 1.09, 1.25). Women who had skin cancer were more likely to undergo cataract surgery (AOR = 1.09, 95% CI = 1.01, 1.17), women who had used hormone replacement therapy were less likely to undergo cataract surgery (AOR = 0.82, 95% CI = 0.69, 0.98) compared to never users, and poor or fair general health (AOR = 1.12, 95% CI = 1.04, 1.22) was associated with higher odds of cataract surgery (Table 2, model 4).

### Sensitivity analysis

We also conducted two separate sensitivity analyses for the effects of different biases or reporting errors. The first sensitivity analysis was performed without excluding prior eye surgery before the commencement of the study (sample size 6792). However, there was no difference in terms of parameter estimates pertaining to factors associated with cataract surgery when compared with the main analysis. The second sensitivity analysis considers the response options as they were reported at each survey without carrying forward prior reports of eye surgery from previous surveys. This analysis will underestimate the proportion of women who had cataract surgery at each survey as it drops prior cataract surgery. For instance, when we look at the proportion of women who underwent cataract surgery across the surveys in this analysis, it was 27.27% in survey four (age 79–84 years), 21.3% in survey five (age 82–87 years), and 14.83% in survey six (age 85–90 years). The effects were similar to the main analysis (data not shown).

## Discussion

### Factors associated with cataract surgery

The aim of this longitudinal study was to assess proportion of and factors associated with cataract surgery in terms of predisposing, enabling, and need factors over 6-year of follow-up. In the study, more than 43.5% of women had undergone eye surgery, including cataracts, and 7.6% had unoperated cataract indicating more than half of the participants either had undergone surgery or had unoperated cataracts (Table 1), which is similar to the Australian Institute of Health Welfare report [8]. The odds of undergoing cataract surgery were significantly increased over time from 1.82 (95% confidence interval 1.73, 1.91) at the age of 82–89 years to 3.39 (95% confidence interval 3.14, 3.66) at the age of 85–90 years compared to baseline 79–84 years of age in the fully adjusted model (Table 2, model 4).

We identified that baseline age and smoking from predisposing factors and private health insurance from enabling factors were positively associated with having cataract surgery. From the need factors, frequent GP visits in the past 12 months of the survey (indicating higher levels of health care need) and skin cancer were positively associated with cataract surgery, while difficulty seeing newspaper print, hormone replacement therapy (HRT), and general health were factors negatively associated with cataract surgery.

### Predisposing factors associated with cataract surgery

Age and smoking were the two variables significantly associated with cataract surgery in the study. In this study, baseline age was significantly associated with cataract surgery, where a 1-year increase in age was 1.11 times more likely significantly associated with cataract surgery after controlling all potential covariates, and this was consistent with previous studies under various settings and different study populations including the Blue Mountains Eye Study among older Australian populations [27, 56, 57]. This may be explained by as a person live longer, prolonged exposure to risk factors, the consequences of ageing resulting in wear and tear, weakened immune system and body defence to repair itself, environmental exposure to ultraviolet radiation from sunlight or occupational hazards, and hereditary predisposition increasing frequent service use that results in cataract and eventually surgery.

In the Australian Longitudinal Study on Women’s Health (ALSWH) population, current or ex-smoker women had 15% higher odds of cataract surgery when compared to women who never smoked in their lifetime. The finding is consistent with many other previous studies, where smoking is associated with cataract as well as cataract surgery [27, 36, 58, 59], and smoking cessation reduced the risk of cataract seems to indicate a causal pathway [36]. In general, smoking is noted to be consistently associated with cataract development and progression.

### Enabling factors associated with cataract surgery

In our study, in the final fully adjusted GEE model, it was revealed that women who have private health cover (insurance) were 1.27 (95% confidence interval 1.16, 1.39) times more likely to undergo cataract surgery compared to those women without private insurance (Table 2, model 4). This is consistent with the prior Australian and international studies, where people who have private health insurance have the opportunity to get cataract surgery done privately when and where they prefer in a private setting [60, 61]. This can be explained by the fact that for public hospitals in which the cost of cataract surgery is covered by Medicare, the waiting time is long, and the priority for treatment is based on disease severity which takes a longer time. Therefore, people who can pay for private health cover and afford the additional out-of-pocket expenses (the gap) can go to a private hospital with the surgeon they prefer and get the surgery sooner, usually as a day procedure. According to a government report, more than 70% of cataract surgery procedure is performed at private facilities in Australia [60, 62].

Regardless of the popular belief in equal access to health services such as cataract surgery in the industrialized countries, such as Australia, the fact is that there is highly unequal access to cataract surgery and similar services in high-income nations [63, 64]. Therefore, serving the poor within the rich should be a focus.

Our finding, in line with other studies [16, 60, 65], may indicate the limited capacity of health system and workforce for increasing demands of cataract surgery due to population ageing at the policy level, socioeconomic inequalities, and lack of timely access to cataract surgery for public-funded Medicare clients at an individual level. This could suggest the need for reform for targeted intervention, equitable, and timely delivery of cataract service.

### Need factors associated with cataract surgery

We identified difficulty seeing newspaper print, general practitioner (GP) visits, hormone replacement therapy (HRT), general health were factors as significantly associated with cataract surgery in the study after controlling several potential confounders for predisposing and enabling factors. In this prospective longitudinal study, we found women who have no difficulty seeing newspaper print were 1.35 times more likely to have had cataract surgery done compared to those with difficulty seeing newspaper print (Table 2, model 4) over time. It has been noted in several previous studies that vision problems, including difficulty seeing newspaper print and the risk of developing cataracts and other ocular diseases, increase with age [3, 27, 66–68]. On the other hand, cataract surgery restores sight remarkably improves visual and cognitive function and vision-related quality of life, as evidenced in many previous studies consistent with our findings [35, 69–73].

Our finding can be explained by the fact that visual function can improve and the expected outcome of cataract surgery; therefore, it is important to timely intervene on unoperated cataract to get the required vision improvement in the very old population as this has a big impact on the independence, vision related, and other aspect quality of life improvement as it enables them to do what they value in their life.

High general practitioner (GP) visit (5 or more times in a year) was 1.16 times more likely associated with cataract surgery in the current study in the final model after controlling potential confounders (Table 2, model 4). A general practitioner (GP) visit is the measure of health service utilization and primary health survives use in many studies [74–77]. GPs are the first point of contact to refer patient as per clinical care standard for cataract recently released [78]*.* In our context, frequent GP visits can be equivalent to cataract surgery service use, so it is not surprising if it has strong association. It can be explained as GP service in the entry point to healthcare use in Australia [77], the first point of contact for many Australians through which they get a referral letter to specialist surgery procedure waiting list [77, 79]. Therefore, the take home-message from this finding could be older peoples visiting their GP for ophthalmologic evaluation due to vision change as a result of ageing or due to any other reason[80] will be more likely to have the opportunity to get cataract surgery service.

The use hormone replacement therapy (HRT) is controversial due to the fact that it may increase the chances of developing some diseases, such as blood clotting or breast cancer when used for longer period [81, 82].

In our study, almost 90% of the women use HRT (Table 1) and it was revealed that women who use HRT were less likely to have undergone cataract surgery compared to never users, the odds ratio was 0.82 (95 per cent confidence interval 0.69–0.98) after controlling numerous potential confounders (Table 2, model 4). This was consistent with some previous studies [82–84], but not all, in which the use of HRT is associated with a lower risk of cataract/ cataract surgery. For example, in The Blue Mountains Eye Study, it was found hormone replacement therapy current users of aged 65 years and above had smaller prevalence of cortical cataract compared to never users [83] as well in the Beaver Dam Eye Study it was revealed that estrogen replacement therapy reduced the risk of cortical cataract [85]. Above all, a metanalysis conducted in 2013 involving nine different studies, four cohort studies, and five case–control and cross-sectional studies that assessed the effect of effects of HRT on cataract for postmenopausal women concluded that HRT had a protective effect on the development of cataract which is in agreement with our finding [84]. On the other hand, several studies also found that HRT was more likely associated with cataract development and the increased risk of cataract surgery, while others did not find an association between HRT and cataract [27, 56, 86, 87]. From our finding, we can conclude that since HRT was less likely associated with cataract surgery and may have protective effect on cataract development and our study strengthens previous findings that the benefit of using HRT outweigh the risk when started within period of 5 years of menopause in women who have no underlying condition, as a review conducted by Barry G Wren revealed [82].

The present study has found that there was a positive association between skin cancer and cataract surgery, where the odds of undergoing cataract surgery were 1.09 times more likely in women who had skin cancer when compared to who never had skin cancer (Table 2, model 4). This finding is novel as only a few studies so far stated the association between skin cancer and cataract among older people to the best of our knowledge [88, 89].

One study, conducted among the Australian population, indicated that cataract was associated with skin cancer among older people 65 years and above [89]. Another study conducted in Israel among persons 40 years and above revealed a positive association between skin cancer from ultraviolet sunlight exposure and cataract [88]. However, both studies were cross-sectional, and no study had assessed the association between skin cancer and cataract or cataract surgery over time. Our study strengthens the previous two cross-sectional studies with the longitudinal data disclosing a positive association between skin cancer and cataract surgery over time. The result may be justified as exposure to sun light may aggravate the progression of age-related cataract due to ultraviolet radiation from sunlight exposure as this is the recognized environmental risk factor for cataract as well skin cancer [68, 88, 90, 91]. Another Australian study also uncovered that the high level of ultraviolet radiation exposure was associated with the rising prevalence of cataracts among native Australians [92], which may strengthen the hypothesis that both skin cancer and cataract might share ultraviolet sunlight exposure as a common causal mechanism [89]. Skin cancer has co-morbidities with other body parts as well as the eye itself, and both skin and eye are the two organs of the body most frequently exposed to sunlight [93].

One previous study identified several ocular side effects of anti-cancer treatment, including dry eye, uveitis, and occlusion of the retinal vein [94]. Ocular side effects of cancer treatment may result in cataract and consequent cataract surgery among our study participants; however, we did not include cancer treatments in our analyses.

In our study, poor general health was associated with increased odds of cataract surgery (Table 2, model 4), potentially reflecting an overall physiological state of diminished intrinsic capacity and, therefore, increased need. Moreover, there is also a risk that waiting times for cataract surgery [16] may increase consequences such as falls, fracture [16, 95], driving cessation [96, 97] and overall social disengagement. Likewise, other factors such as depression and cognitive decline could explain the association between poor general health and cataract surgery, as evidenced in a previous study [98]. Accordingly, general health may be compromised affecting quality of life.

The association between surgery and not having poor vision (difficulty seeing newspaper print) may reflect the corrective effects of the surgery, restoring the close vision of those who had been able to access the procedure. Previous studies have reported inconsistent associations between self-rated health and cataract surgery, where some studies found no association particularly for people older than 80 years [34]. For instance, in the Blue Mountains Eye Study, researchers didn’t find statistically significant association between change in general health and cataract surgery over 10-year follow-up [34]. On the other hand, other studies reported positive association between general health and cataract surgery or cataract as well [99, 100].

There were some limitations in this study. A major limitation is that cataract surgery was ascertained through self-report, and within a question about eye surgery more generally. However, we would expect that cataract removal would be the bulk of these surgeries. We also couldn’t identify the different techniques of cataract surgery performed. We don’t have data for ultraviolent radiation exposure from sun light which is a well-known risk factor for age-related cataract and then cataract surgery. Instead, we must rely on the association between skin cancer and cataract surgery, as a proxy for sun exposure and perhaps also the mitigating effects of skin pigmentation. Strengths include the longitudinal nature of the data, over 6-year of follow-up, the large and representative sample from all Australian states and Territories. The study is thus not limited to specific geographic areas as previous Australian studies have been [56, 99, 101, 102].

## Conclusion

The odds of undergoing cataract surgery were significantly increased with time over 6 years of follow-up as the women aged. Increasing age and smoking were predisposing factors positively associated with cataract surgery. Having private health insurance was associated with undergoing cataract surgery from enabling factors.

Frequent GP visits and skin cancer were also positively associated with cataract surgery, while difficulty seeing newspaper print, hormone replacement therapy, and general health factors negatively associated with cataract surgery. These need factors were the major drivers of cataract surgery utilisation; however, predisposing and enabling factors also play a role, including access to private health insurance among older Australian women in their 80 s. This finding indicates some inequity in relation to access to cataract surgery in the Australian setting, which may be highly important for women’s social engagement and wellbeing that policymakers need to target.

## Data Availability

ALSWH data set use is available only for approved collaborating researchers which is subject to rigorous ethical requirements because of the sensitive personal nature of the collected data based on formal request to make use of the data. Additional details are available at http://alswh.org.au/for-researchers.

## Code availability

Codes will be made available upon reasonable request.

## Declarations

### Statement of human and animal right

The ALSWH project has continuous ethical approval from the Human Research Ethics Committees (HREC) of the University of Newcastle and the University of Queensland, Australia.
